# Lung-protective effect of Punicalagin on LPS-induced acute lung injury in mice

**DOI:** 10.1042/BSR20212196

**Published:** 2022-01-24

**Authors:** Yibin Zeng, Hongying Zhao, Tong Zhang, Chao Zhang, Yanni He, Lingbo Du, Fuguo Zuo, Wuqing Wang

**Affiliations:** 1 Department of Dermatology, Minhang Hospital, Fudan University/Central Hospital of Minhang District, Shanghai 201199, China; 2 Department of Dermatology, Chinese Medicine Hospital in Juxian, Shandong Province 276500, China; 3 Department of Dermatology, East Hospital, School of Medicine, Tongji University, Shanghai 200120, China

**Keywords:** Acute lung injury, Lipopolysaccharide, MAPK, NF-κB, Punicalagin

## Abstract

**Background:** Punicalagin (Pun) is one of the main bioactive compounds in pomegranate peel, it possesses many properties, including antioxidant, anti-inflammation and immunosuppressive activities. The study was aimed to investigate the protective effect and mechanisms of Pun on lipopolysaccharide (LPS)-induced acute lung injury (ALI) in mice.

**Methods and Results:** Forty-eight BALB/c male mice were used to establish ALI by intratracheal-instilled 2.4 mg/kg LPS, the mice were randomly divided into model and Pun (10, 20, 40 mg/kg) groups. The other 12 mice were intratracheal-instilled same volume of water as control. After 2 h of receiving LPS, mice were administered drug through intraperitoneal injection. Lung index, histopathological changes, white blood cells and biomarkers in bronchoalveolar lavage fluid (BALF) were analyzed. The protein expression of total and phosphor p65, IκBα, ERK1/2, JNK and p38 in lung tissue was detected. The result showed that Pun could reduce the lung index and wet/dry weight (W/D) ratio, improve lung histopathological injury. In addition, Pun decreased the inflammation cells and regulated the biomarkers in BALF. Furthermore, Pun dose-dependently reduced the phosphor protein levels of p65, IκBα, ERK1/2, JNK and p38 in lung tissue, which exhibited that the effect of Pun related to mitogen-activated protein kinases (MAPKs) pathway. More importantly, there was no toxicity was observed in the acute toxicity study of Pun.

**Conclusion:** Pun improves LPS-induced ALI mainly through its anti-inflammatory properties, which is associated with nuclear factor-κB (NF-κB) and MAPKs signaling pathways. The study implied that Pun maybe a potent agent against ALI in future clinic.

## Introduction

Acute lung injury (ALI) is a clinical syndrome caused by pathological changes in lung tissue by various factors [1]. The main pathological features are diffuse pulmonary damage, increased permeability of capillary basement membrane, massive protein-rich edema fluid infiltrating into pulmonary interstitium or alveoli [2,3]. The main clinical manifestations were severe respiratory distress and intractable hypoxemia. Clinically, the morbidity is very high, and the mortality rate is as high as 50% [3]. Acute lung injury/acute respiratory distress syndrome (ALI/ARDS) is an acute, progressive respiratory disorder or respiratory failure caused by a variety of cardiogenic and extraneous factors such as trauma, infection, shock and diffuse coagulation in blood vessels [4,5]. The essence of the disease is a syndrome caused by systemic inflammatory response [4,5]. The pathophysiological changes in ALI and ARDS are similar, but they are at different stages, and ARDS is more serious than ALI. ARDS occurs rapidly and progresses rapidly, thus early detection, early treatment and early intervention are emphasized in the treatment of ARDS [6]. At present, although a variety of clinical treatment measures have been taken, no effective treatment has been achieved, and its pathogenesis has not yet been fully clarified. Therefore, it is urgent to find effective therapeutic drugs.

**Figure 1:**
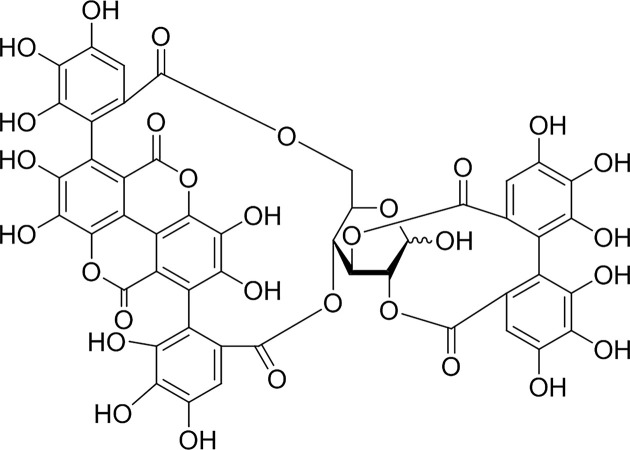
Chemical structure of Pun

*Punica granatum* L. (Pomegranate), a healing food, has been known for hundreds of years. Pomegranate and its peel have many pharmacological properties, which were widely used in folk medicine for the treatment of respiratory diseases, diarrhea, parasite infection, hemorrhage and ulcers [7–10]. Punicalagin (Pun, 2,3-hexahydroxy diphenoylgallagyl-d-glucose, Figure 1) is one of the main active tannins isolated from *P. granatum* L. fruit and *Terminalia catappa* leaves. It is the largest ellagitannin and polyphenolic antioxidant molecule with water solubility and high bioavailability. Pun was proved to be an important bioactive constituent in pomegranate peel, which has been reported to possess antioxidant, reduces oxidative stress, anti-inflammation, anti-proliferation, apoptosis, immunosuppressive activities [11–14], and also shows hepatoprotective activity in rats [15].

In addition, current research reported that in *in vitro* experiments, Pun could attenuate mitochondrial dysfunction and inflammatory responses, which exert beneficial functions in 6-hydroxydopamine-treated SH-SY5Y cells [16]. Seok et al. reported that Pun could rescue cell viability and attenuate inflammatory responses of human epidermal keratinocytes, exposed to airborne particulate matter PM10 [17]. In *in vivo* animal experiment, Pun exhibited decreased serum glucose levels, increased PON1 activity and HDL anti-inflammatory values in BALB/c mice with a high-fat diet [18]. Wang et al. reported that Pun could reduce the oxidative stress marker, restore the angiogenic balance in a rat model of pregnancy-induced hypertension [19]. Furthermore, Pun also displayed that it can suppress the obesity and obesity-induced inflammatory responses through the Nrf2/Keap1 signaling pathway [20]. The work of El-Beih et al. showed that Pun had a protective effect on some key regulators of insulin resistance and oxidative liver injury in streptozotocin-nicotinamide type 2 diabetic rats [21].

Above all, to the best of our knowledge, although Pun has many pharmacological activities, the information about its effects on ALI is limited in the literature. Therefore, in the present study, we aimed to investigate the protective effect and elucidate the possible potential mechanisms of Pun on lipopolysaccharide (LPS)-induced ALI in mice. The results of the study would clarify the properties of Pun on ALI and provide a theoretical basis for its future potential use in clinic.

## Materials and methods

### Materials and reagents

Pun (>90%) was purchased from Beijing Huanyu Kechuang Biological Company (Beijing, China). LPS (*Escherichia coli* O55:B5) was purchased from Sigma–Aldrich (Darmstadt, German). Mouse tumor necrosis factor-α (TNF-*α*, Batch No 978941030), interleukin (IL)-10 (IL-10, Batch No 132111031116), IL-6, (Batch No 1321213119) and transforming growth factor-β1 (TGF-β1, Batch No 9711891510) were purchased from Wuhan Boster Biology Technology Co. Ltd. (Wuhan, China). Total-antioxidant capacity (T-AOC; Batch No A024) and malondialdehyde (MDA; Batch No A003-1) were purchased from Nanjing Jiancheng Biotechnology Company (Nanjing, China). Mouse anti-human nuclear factor-κB p65 (NF-κB p65, #8242), phosphorylated nuclear factor-κB p65 (pNF-κB p65, #3033), IκBα (#9242), phosphor IκBα (#2859), ERK1/2 (#9102) and phosphor ERK1/2 (#9101) were purchased from Cell Signaling Technology; JNK (sc-7345), phosphor JNK (sc-6254), p38 (sc-7972) and phosphor p38 (sc-7973) antibody were purchased from Santa Cruz (CA, U.S.A.). β-actin antibody was purchased from Sungen (China). Anti-rabbit IgG, HRP-linked antibody (7074S) and anti-mouse IgG, HRP-linked antibody (7076S) were purchased from Cell Signaling Technology. Enhanced chemiluminescence detection kit was obtained from Merck Millipore (Darmstadt, Germany).

### Animals

The male BALB/c mice with body weight 20 ± 2 g and age: 8 weeks were provided by the animal experimental center of the Fudan University (Shanghai, China). Six mice were raised in one polyacrylic cage, and all the mice were quarantined for 1 week before use. All the mice were reared in the animal experimental center of Fudan University in SPF grade environment with free access to food and water (24 ± 1°C, 50 ± 5% of humidity and 12-h day/night cycle). The mice received human care in the terms of National Institutes of Health Guidelines, U.S.A. (National Research Council of U.S.A., 1996) and the university ethical regulations of Fudan University.

### Experimental design

Sixty male BALB/c mice with the age of 8 weeks (22 ±2 g) were randomly divided into five groups (*n*=12): control group, model group, Pun 10 mg/kg, Pun 20 mg/kg and Pun 40 mg/kg groups. The mice in model and Pun treated groups were intratracheal-instilled with 2.4 mg/kg LPS in saline under the anesthesia using sodium pentobarbital (40 mg/kg). The mice in control group were intratracheal-instilled with saline. After receiving LPS 2 h, the mice were administrated with Pun (10, 20, 40 mg/kg) or saline (control and model group) through intraperitoneal injection, respectively. After 12 h of drug treatment, all the mice were executed by cervical dislocation. Then, the bronchoalveolar lavage fluid (BALF) and lung tissue for biomarker detection were harvested.

Forty male BALB/c mice with the age of 8 weeks (22 ± 2 g) were used for acute toxicity study. All the mice were randomly divided into four groups (*n*=10): Control group, Pun 100 mg/kg, Pun 500 mg/kg and Pun 5000 mg/kg groups. All the group mice except control group were orally administered with Pun 100 mg/kg, Pun 500 mg/kg and Pun 5000 mg/kg, respectively. The control group mice were orally administered with saline. After drug treatment, all the mice were fed normally, their mental status and survival were observed for 14 days. All animal studies were carried out at the animal center of Fudan University, and all the experiment protocols were approved by the animal care and use committee of Fudan University with the approval number YZ201905.

### Histopathological analysis

Right lower lobe of lung tissue in each group was fixed in the 40 g/l formaldehyde solution overnight. The fixed lung tissues were embedded in paraffin, cut into 5-µm-thick sections and then stained with Hematoxylin and Eosin (H&E) in terms of the routine histopathological examination. The final stained sections were photographed under a light microscope (BX-50 Olympus) at 200× magnification.

### Effect of Pun on lung wet/dry weight ratio and lung index

At the end of the experiment, the body weight of mice was collected, lung tissues in all groups were harvested, the surface water of lung with filter paper was absorbed and the lung was weighed by precise electronic balance, the data were named wet weight of lung. Then the lung was completely dried in an oven at 80°C for 48 h until the weight of lung tissue did not change, the dry lung was weighed and the data were named dry weight of lung. Then the ratio of wet/dry weight (W/D) of lung tissue in mice was calculated in order to evaluate the degree of pulmonary edema. In addition, lung index (lung index % = wet lung weight/body weight × 100) was calculated to further confirm the degree of pulmonary edema.

### Harvesting the BALF and the cell numbers statistics

At the end of the experiment, mice were killed by cervical dislocation and fixed on the operating table. They were then disinfected by alcohol and the trachea was fully exposed, 18G trocar was inserted and fixed with silk thread. The chest of mice was opened to expose the lung tissue, and the right lung was ligated with silk thread. The left lung was slowly lavaged with 0.5 ml PBS at 4°C. The lavage was repeated three times, and withdrawn 30 s per time. The lavage solution was collected and transferred to the centrifugal tube, centrifuged at 4°C, 700×***g*** for 5 min. The supernatants were harvested into the sterilized EP tubes, and stored at −80°C for use. Then the precipitated cells were suspended with 50 μl PBS, and stained with Diff-Quik stain. The total cells, neutrophils and macrophages in BALFs of mice in all the group were counted by cell counting plate under optical microscope (300 cells per smear).

### The levels of TNF-*α*, IL-6, IL-10 and TGF-β1 in BALF detection

The levels of TNF-*α*, IL-6, IL-10 and TGF-β1 in BALF of mice in all the group were detected according to the instructions of ELISA kit (Wuhan Boster Biology Technology co. ltd., Wuhan, China).

### The activity of T-AOC and the contents of MDA in lung tissue assay

The lung tissue homogenate was prepared by electric grinder: 50 mg of lung tissue with 200 μl cold RIPA lysis buffer (P0013B, Beyotime) was added, and homogenized in ice bath. Then centrifuged at 4°C, 10000 rpm for 5 min. The supernatants were collected, and measured the protein concentration with BCA protein assay kit (71285-3, Merk). The activity of T-AOC and the contents of MDA were detected according to the kit protocol from Nanjing Jiancheng Biotechnology Company (Nanjing, China).

### Western blot assay

The protein expression of total NF-κB p65 (dilution ration 1:1000), IκBα (1:1000), ERK1/2 (1:1000), JNK (1:2000), p38 (1:2000) and phosphor NF-κB p65 (1:1000), IκBα (1:1000), ERK1/2 (1:1000), JNK (1:2000), p38 (1:2000) in lung tissue were assayed by Western blot. First, the lysate of lung tissues were prepared: 50 mg of lung tissue with 200 μl of cold RIPA lysis buffer, homogenized in ice bath, centrifuged at 4°C, 10000 rpm for 5 min. The supernatants were harvested and measured the protein concentration with BCA protein assay kit, then 5× SDS loading buffer was added, boiled for 5 min at 95°C. Then, 20 μg of protein samples were taken to load at 12% SDS/PAGE electrophoresis gel for running. Then the protein was transferred from gel to PVDF membrane at 110 V for 1.5 h on ice bath. The membrane were then blocked with 5% FBS solution at room temperature for ∼1 h. The primary antibody was diluted with 5% FBS solution, and incubated the PVDF membrane under the diluted primary antibody at 4°C for overnight (∼12 h). Then the membrane was washed with 1× TBST solution for 10 min at room temperature, and repeated three times. The second antibody of anti-rabbit (1:10000) and anti-mouse (1:5000) were diluted with 1× TBST solution, and incubated the membrane at room temperature for 1 h. The membrane was washed with 1× TBST solution for 10 min at room temperature, and repeated three times. Finally, the membrane was imaged at Syngene GBO X gel scan imager after ECL reagent visualization.

### Statistical analysis

Values were represented as mean ± standard deviation (SD). All statistical comparisons were calculated by means of a one-way ANOVA test followed by Dunett’s *t* test with SPSS19.0 statistical software (IBM Corporation, Armonk, NY, U.S.A.). All experiments were performed at least three times independently. *P*<0.05 was regarded as statistically significant.

## Results

### General situation and survival observation of all the mice

At the end of the experiment, all mice were alive. The mice in control group had a high activity and normal diet. The mice in model group had a general activity and reduced food intake. The behavior of the mice in Pun-treated groups was basically between the control group and the model group, it was better than the model group mice, and a little worse than the control group mice, especially the low-dose Pun-treated group.

In the acute toxicity study, after 14-day observation, all the mice were alive, and had a high activity and normal diet. Through anatomical observation, all organs including lung, liver, spleen, kidney, stomach, intestine and heart did not observe morphological changes. In addition, there were no significant differences in body weight between drug administered group mice and control group mice.

### Effect of Pun on the number of total cells, neutrophils and macrophages in BALF of ALI mice

From Table 1, we could see that, compared with the control group, the number of the total cells, neutrophils and macrophages in BALF were significantly increased in model group (*P*<00.01). While after Pun treatment, the number of total cells, neutrophils and macrophages in BALF of ALI mice were dramatically decreased, compared with model group (*P*<0.05, *P*<0.01), especially in the high-dose (40 mg/kg) Pun-treated group (*P*<0.01). These results suggest that Pun can reduce the number of total cells, neutrophils and macrophages in BALF of LPS-induced ALI mice in a dose-dependent manner (Table 1).

**Table 1:** Number of total cells, neutrophils and macrophages in BALF of mice in each group

Group	Total cells (×10^7^/ml)	Neutrophils (×10^7^/ml)	Macrophages (×10^6^/ml)
Control	2.21 ± 0.13	0.54 ± 0.13	1.06 ± 0.12
Model	4.47 ± 0.23^1^	3.46 ± 0.44^1^	3.41 ± 0.40^1^
Pun 10 mg/kg	3.34 ± 0.26^2^	2.83 ± 0.72^2^	2.70 ± 0.35^2^
Pun 20 mg/kg	2.95 ± 0.41^3^	2.04 ± 0.61^3^	2.21 ± 0.23^3^
Pun 40 mg/kg	2.14 ± 0.22^3^	1.21 ± 0.43^3^	1.16 ± 0.10^3^

Data are presented as mean ± SD (*n*=12). ^1^*P*<0.01 *vs* control group. ^2^*P*<0.05. ^3^*P*<0.01 *vs* model group.

### Effect of Pun on the levels of TNF-*α*, IL-6, IL-10 and TGF-β1 in BALF of ALI mice

Since the inflammation in ALI disease, here, we detected the levels of pro-inflammatory factors of TNF-*α* and IL-6, and the anti-inflammatory factors of IL-10 and TGF-β1 by ELISA. The results showed that the levels of TNF-*α* and IL-6 in model group were significantly increased, while the levels of IL-10 and TGF-β1 were dramatically decreased in model group, compared with the control group (Table 2, *P*<0.01). After Pun (10, 20 and 40 mg/kg) treatment, the levels of TNF-*α* and IL-6 in BALF were significantly reduced, and the levels of IL-10 and TGF-β1 in BALF were increased compared with the model group (Table 2, *P*<0.05, *P*<0.01). The result exhibited that Pun can decrease the levels of TNF-α and IL-6, increase the levels of IL-10 and TGF-β1 in BALF, which suggested that Pun has an anti-inflammatory effect in ALI mice.

**Table 2:** Effect of Pun on the levels of TNF-*α*, IL-6, IL-10 and TGF-β1 in BALF

Group	TNF-*α* (ng/ml)	IL-6 (ng/ml)	IL-10 (ng/ml)	TGF-β1 (ng/ml)
Control	0.37 ± 0.01	0.11 ± 0.03	0.41 ± 0.10	0.36 ± 0.10
Model	1.87 ± 0.23^1^	1.44 ± 0.20^1^	0.06 ± 0.01^1^	0.08 ± 0.01^1^
Pun 10 mg/kg	1.43 ± 0.21^2^	1.03 ± 0.10^2^	0.09 ± 0.10^2^	0.09 ± 0.10^2^
Pun 20 mg/kg	1.01 ± 0.11^3^	0.87 ± 0.10^3^	0.18 ± 0.05^3^	0.18 ± 0.13^3^
Pun 40 mg/kg	0.54 ± 0.09^3^	0.32 ± 0.07^3^	0.40 ± 0.11^3^	0.29 ± 0.05^3^

Data are presented as mean ± SD (*n*=12). ^1^*P*<0.01 *vs* control group. ^2^*P*<0.05. ^3^*P*<0.01 *vs* model group.

### Effect of Pun on the activity of T-AOC and the content of MDA in lung tissue of ALI mice

Due to anti-inflammatory effect of Pun showed above, we continued detection of the antioxidant activity of Pun. From Table 3, we can see that the activity of T-AOC was significantly decreased in model group, compared with the control group (Table 3, *P*<0.01). After Pun treatment, the levels of T-AOC were markedly increased, compared with the model group (*P*<0.05, *P*<0.01). In addition, the content of MDA in model group was significantly increased, compared with the control group (Table 3, *P*<0.01). Pun significantly decreased the content of MDA in three dosages of treated group, compared with the model group (*P*<0.05, *P*<0.01). The results showed that Pun possessed the antioxidant activities and also can reduce the lipid peroxidant products in a dose-dependent manner.

**Table 3:** Effect of Pun on the levels of T-AOC and MDA in lung tissues

Group	T-AOC (U/mg)	MDA (nmol/mg)
Control	267.35 ± 20.52	3.64 ± 1.30
Model	123.16 ± 15.34^1^	9.03 ± 1.27^1^
Pun 10 mg/kg	175.20 ± 11.26^2^	7.55 ± 1.05^2^
Pun 20 mg/kg	198.75 ± 10.41^3^	5.41 ± 1.15^3^
Pun 40 mg/kg	231.70 ± 17.69^3^	4.37 ± 1.22^3^

Data are presented as mean ± SD (*n*=12). ^1^*P*<0.01 *vs* control group. ^2^*P*<0.05. ^3^*P*<0.01 *vs* model group.

### Effect of Pun on the lung W/D ratio and lung index of ALI mice

**Figure 2:**
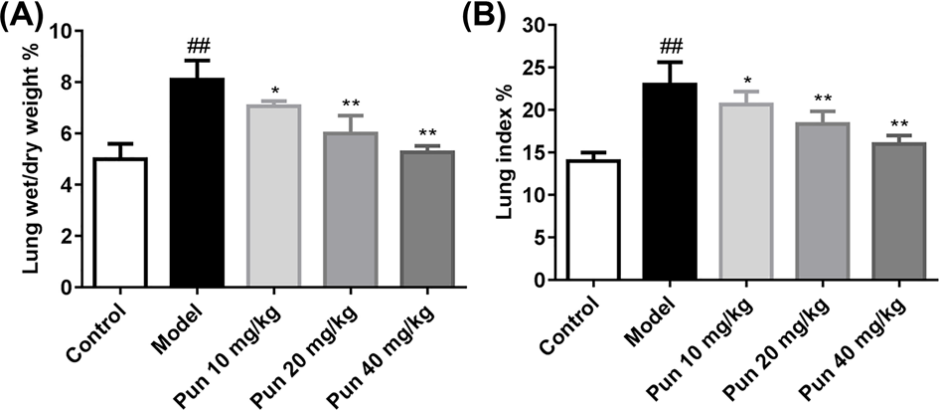
The lung W/D ratio and lung index in ALI mice (*n*=12) (**A**) The values of lung W/D of each group. (**B**) Lung index of each group (lung index % = wet lung weight/body weight × 100). ^##^*P*<0.01 *vs* control group, *P<0.05, **P<0.01 *vs* model group.

In order to detect the effect of Pun on pulmonary edema, we assayed the lung (/D)ratio and lung index. Compared with the control group, the W/D ratio and lung index in model group were significantly increased (Figure 2, *P*<0.01). Compared with the model group, Pun (10, 20 and 30 mg/kg) treated groups dramatically reduced the elevate W/D ratio and lung index (Figure 2, *P*<0.05, *P*<0.01) in a dose-dependent manner. The results displayed that Pun treatment can relieve the degree of pulmonary edema in ALI mice.

### Effect of Pun on lung histopathology in ALI mice

**Figure 3:**
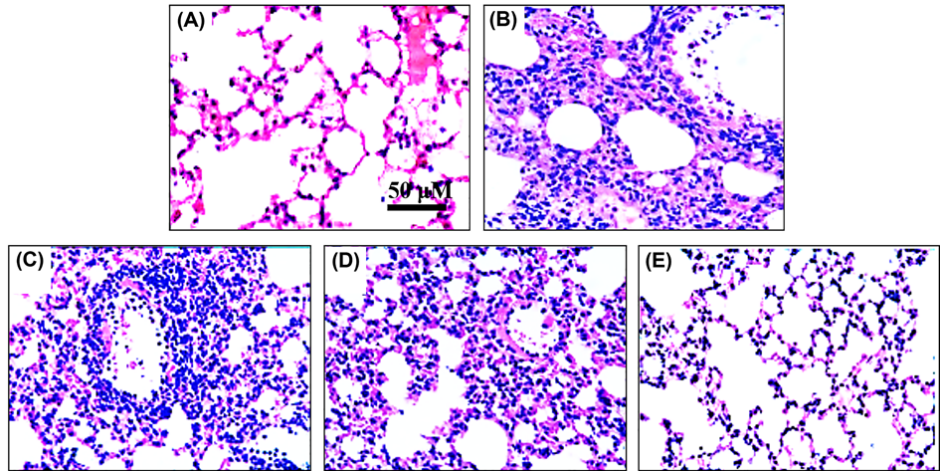
The lung histopathology images of lung tissue for ALI mice (H&E staining, scale bar 50 μm) (**A**) Control group, (**B**) Model group, (**C**) Pun 10 mg/kg, (**D**) Pun 20 mg/kg, (**E**) Pun 40 mg/kg.

Furthermore, we assayed the changes of lung histopathology in each group. From Figure 3A, we can see that the structure of lung lobules in control group was intact, normal alveolar wall, no alveolar cavity exudation, no inflammatory cell infiltration, no transparent membrane formation. In Figure 3B of the model group, there showed a large number of infiltrated inflammatory cells in lung tissue, the alveolar septum became thicker, the transparent membrane was formed, alveolar cavity exuded, pulmonary interstitial edema, the alveolar structure was destroyed and bleeding focus was found. While from Figure 3C–E, we can see that the interventions of Pun at the doses of 10, 20 and 40 mg/kg could remarkably alleviate the pathological damage of lung in ALI mice, especially in the high dose of Pun.

### Effect of Pun on NF-κB signaling pathway in ALI mice

**Figure 4:**
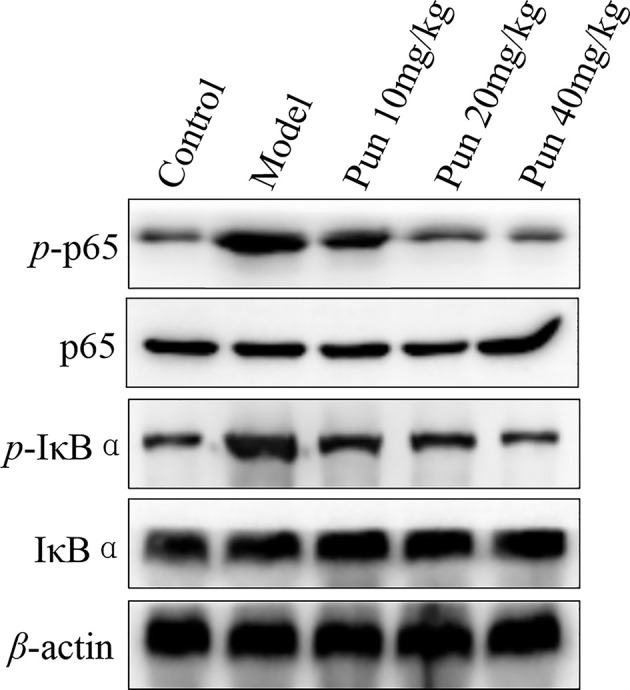
The protein expression of total and phosphor p65 and IκBα in lung tissue The data were repeated by three independent experiments.

Next, we further detected the influence of Pun on NF-κB signaling pathway in ALI mice (Figure 4 and Supplementary Figures S1–S5). The result showed that the levels of phosphorylation NF-κB p65 and phosphorylation IκBα in lung tissue were significantly increased in the model ALI mice, compared with the control group (Figure 4, *P*<0.01). After Pun treatment, the protein expression of phosphorylation NF-κB p65 and phosphorylation IκBα were apparently reduced, compared with the model group, especially in the high-dose Pun group (Figure 4, *P*<0.01). This result exhibited that the effect of Pun on ALI mice related with the NF-κB signaling pathway.

### Effect of Pun on mitogen-activated protein kinases signaling pathway in ALI mice

**Figure 5:**
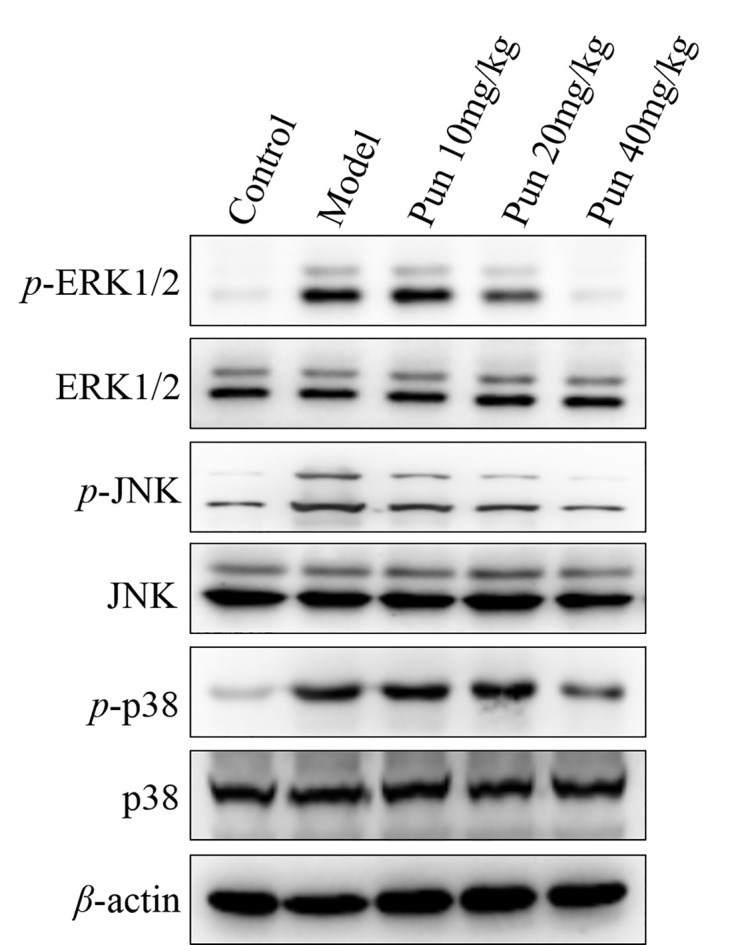
The protein expression of the total and phosphor ERK 1/2, JNK and p38 in lung tissue The data were repeated by three independent experiments.

In addition, based on the NF-κB signaling pathway, we continue detection of the mitogen-activated protein kinases (MAPKs) pathway (Figure 5 and Supplementary Figures S6–S12). The levels of phosphorylation ERK1/2, phosphorylation JNK and phosphorylation p38 in lung tissue of ALI mice were detected. The results showed that the levels of the phosphorylation ERK1/2, phosphorylation JNK and phosphorylation p38 in lung tissue were markedly increased in the model group, compared with the control group (Figure 5, *P*<0.01). Pun-treated group (10, 20 and 40 mg/kg) showed a reducing expression of phosphorylation of ERK1/2, phosphorylation of JNK and phosphorylation of p38, compared with the model group, especially in the high-dose Pun group (Figure 5, *P*<0.05 and *P*<0.01). These results suggested that Pun alleviate the ALI mice related with the MAPKs signaling pathway.

## Discussion

ALI is a kind of respiratory tract infectious disease characterized by neutrophil infiltration, destruction of the integrity of endothelial cells and alveolar epithelial cells, and diffuse injury of alveolar capillaries [22]. The clinical manifestations of ALI are pulmonary edema and acute respiratory distress [23]. LPS is a major bioactive component of the cell wall of Gram-negative bacteria and has been widely used to induce the pulmonary inflammation in ALI mice for a long time [24]. Because of the small size, rapid growth and simple source, the mouse model of ALI induced by LPS through intranasal perfusion is a practical and effective tool in the experimental study of ALI.

Pulmonary edema is the central link of the pathogenesis in ALI. In this experiment, the degree of pulmonary edema was evaluated by measuring the W/D ratio of lung and lung index [25]. The results showed that Pun could significantly inhibit the development of pulmonary edema in ALI mice. Neutrophils play an important role in the pathogenesis of ALI. The previous studies showed that removal of neutrophils can reduce the damage of ALI. In the study, we found that Pun could significantly decrease the numbers of total white cells, the neutrophils and the macrophages in BALF, especially in the high-dose Pun-treated group (40 mg/kg).

Cytokine is a low molecular weight soluble glycoprotein, secreted by various tissues and cells [26]. Cytokine is an important inflammatory mediator in inflammatory response [27]. It can regulate innate immune response and participate in specific immune responses. Studies have confirmed that both pro-inflammatory (IL-6, TNF-*α*) and anti-inflammatory cytokines (IL-10, TGF-β1) play an important role in the development of ALI [28]. LPS, as the main component of the cell wall of Gram-negative bacteria, has the function of activating macrophages *in vivo* and inducing multiple active factors from target cells [29]. When LPS acts on the body, TLR4 signal transduction pathway is activated firstly, which promotes the expression of inflammatory factors of TNF-*α*, IL-6, IL-1β, and interferon (IFN), to induce strong inflammatory response, increases the activity of downstream NF-κB and MAPKs [30]. In the study, Pun significantly reduced the levels of pro-inflammatory cytokines (IL-6 and TNF-*α*), and at the same time, Pun also remarkably increased the levels of anti-inflammatory cytokines (IL-10 and TGF-β1), which reflected that Pun can regulate the inflammation cytokines in order to protect the lung injury.

NF-κB is a kind of nuclear transcription factor existing in the form of homologous and heterodimer, mainly composed of P50 and P65 proteins. It has been found that NF-κB can participate in the regulation of inflammatory response and inflammatory cytokines, and play an extremely important role in the body’s defense and resistance to inflammatory response [27]. IκBα is one of the important member of IκB family, which plays an important role in NF-κB signaling pathway, and mainly regulates the activation and transcription of NF-κB. In the present study, we found that treatment with Pun can significantly reduce the protein levels of phosphor p65 and IκBα in lung tissue, which means that the effect of Pun on lung injury was associated with the NF-κB signaling pathway.

MAPK family is relatively conservative in evolution and has the function of regulating cell differentiation, proliferation and death. Various subtypes have been identified, such as ERK1/2, JNK and p38 [31]. Studies have shown that endotoxin can activate the MAPK signaling pathway, regulate cytokines TNF-*α* and IL-6, recruit and activate white blood cells through the production and activation of inflammatory mediators. These cascades of inflammation induced by activation of inflammatory signaling pathways promote the occurrence and development of ALI [32]. While in our study, we found that Pun can reduce the protein levels of phosphor ERK1/2, phosphor JNK and phosphor p38 in lung tissue, which implied that the effect of Pun involved in the signaling pathway of MAPK.

In the present study, we explored the effects and the potential mechanisms of Pun in ALI mice *in vivo*. The result of the study showed that Pun can significantly reduce the expression of phosphor NF-κB p65, phosphor IκBα, phosphor ERK1/2, phosphor JNK and phosphor p38 in lung tissue. In addition, Pun can also dramatically decrease the cytokine levels of the pro-inflammation (TNF-*α* and IL-6), and increase the cytokine levels of anti-inflammation (IL-10 and TGF-β1). Furthermore, Pun also alleviate the histopathological injury of lung in ALI mice. The current results exhibited that the effect of Pun on ALI maybe associated with the signaling pathway of NF-κB and MAPK, while the cross-talk between these two signaling pathways, and the direct mechanism/action target of Pun in ALI are the underway work in our laboratory, which will be reported in due course.

In conclusion, Pun showed a significant protective effect on ALI mice, it can reduce the levels of inflammation cytokine, alleviate the lung histopathology, reduce the white blood cells and enhance the antioxidant capacity, mainly related with the influence on the signaling pathway of NF-κB and MAPKs.

## Supplementary Material

Supplementary Figures S1-S12

## Data Availability

The datasets used and/or analyzed during the current study are available from the corresponding author on reasonable request.

## Abbreviations

ALI: acute lung injury
ARDS: acute respiratory distress syndrome
BALF: bronchoalveolar lavage fluid
IL: interleukin
LPS: lipopolysaccharide
MAPK: mitogen-activated protein kinase
MDA: malondialdehyde
NF-κB p65: nuclear factor-κB p65
Pun: Punicalagin
SPF: specific pathogen free
T-AOC: total-antioxidant capacity
TGF-β1: transforming growth factor-β1
TNF-α: tumor necrosis factor-α
W/D: wet/dry weight
