# Supplementary material for: Lung-protective effect of Punicalagin on LPS-induced acute lung injury in mice
Source: Biosci Rep. 2022 Jan 24;42(1):BSR20212196. doi: 10.1042/BSR20212196 (PMC8787312; doi:10.1042/BSR20212196)
Supplement: Supplementary Figures S1-S12 [file BSR-2021-2196_supp.pdf]

Lung protective effect of Punicalagin on LPS-induced acute lung injury in mice

Yibin Zeng[1#], Hongying Zhao[2#], Tong Zhang[1], Chao Zhang[1], Yanni He[1], Lingbo Du[1], Fuguo Zuo[3*], Wuqing Wang[1*]

[1] Department of Dermatology, Minhang Hospital, Fudan University/Central Hospital of Minhang District, Shanghai, 201199, China

[2] Department of Dermatology, Chinese Medicine Hospital in Juxian, Shandong Province, 276500, China

[3] Department of Dermatology, East Hospital, School of Medicine, Tongji University, Shanghai 200120, China

[#]The author contributed equally.

[*]The author are co-corresponding author.

Yibin Zeng: mitangbaba@163.com；

Hongying Zhao: zhy19880526@126.com；

Tong Zhang: zhangtongnao@sina.com；

Chao Zhang: charleszhang1980@126.com；

Yanni He: heyanni1978@163.com；

Lingbo Du: dulingbo07@139.com；

Fuguo Zuo: zfg5747@126.com；

Wuqing Wang: wuqing_wang@fudan.edu.cn

[*] Corresponding Author: Wuqing Wang, Department of Dermatology, Minhang Hospital, Fudan University, 170 xinsong road, Minhang District, Shanghai, 201199, China. Telephone: 86-021-38804518. E-mail: wuqing_wang@fudan.edu.cn

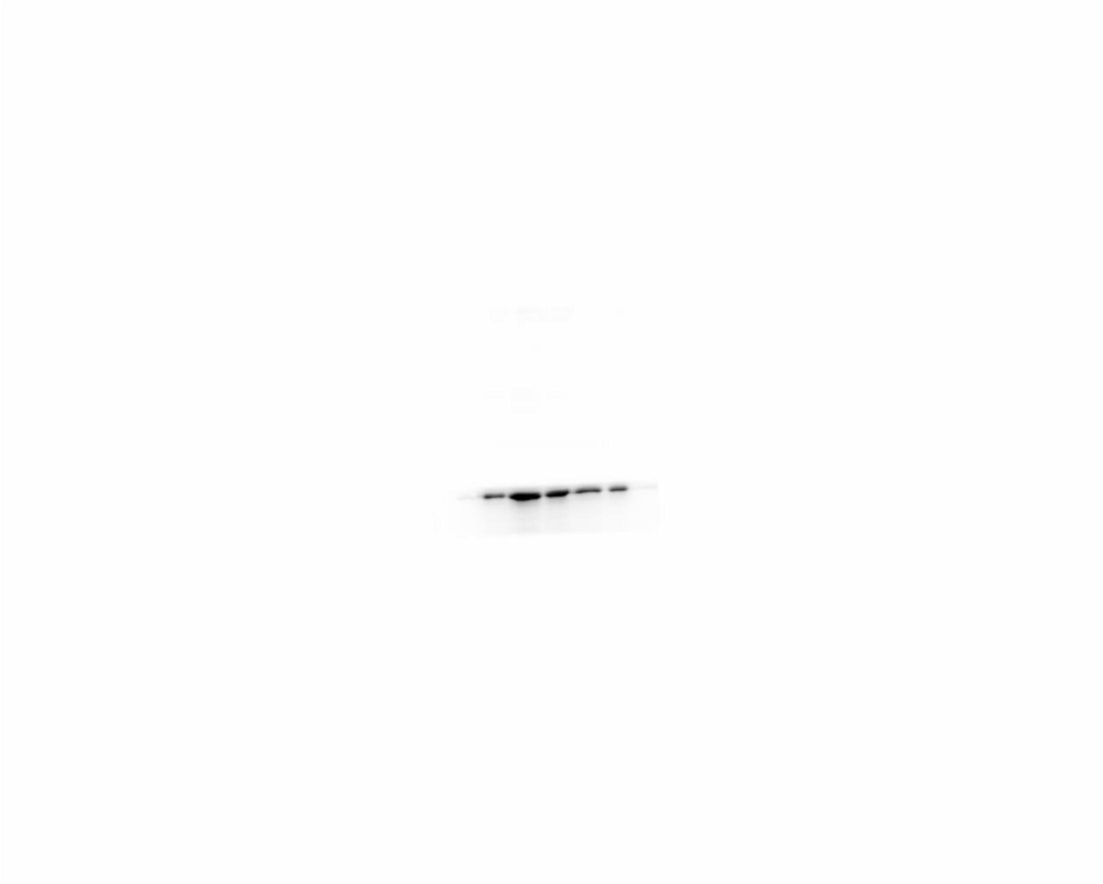

Figure S1 Un-cropped p-p65.

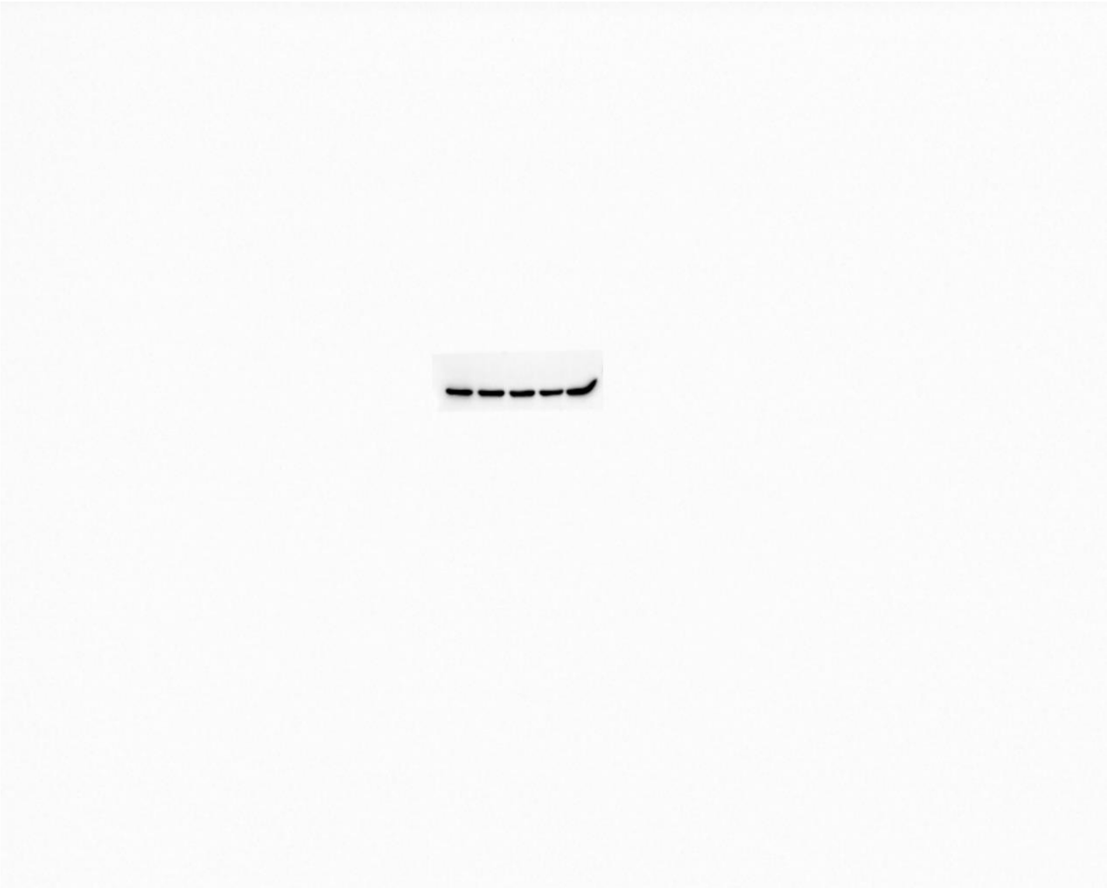

Figure S2 Un-cropped p65.

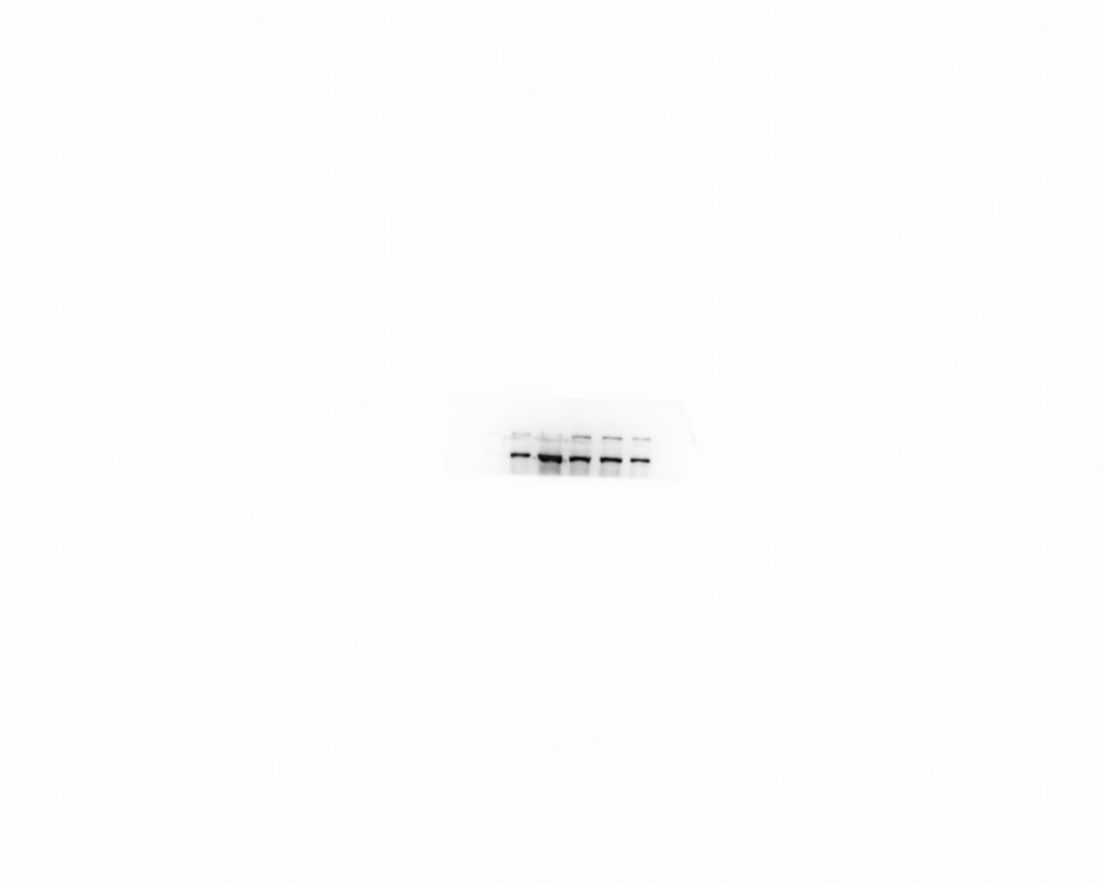

Figure S3 Un-cropped p-IκBα.

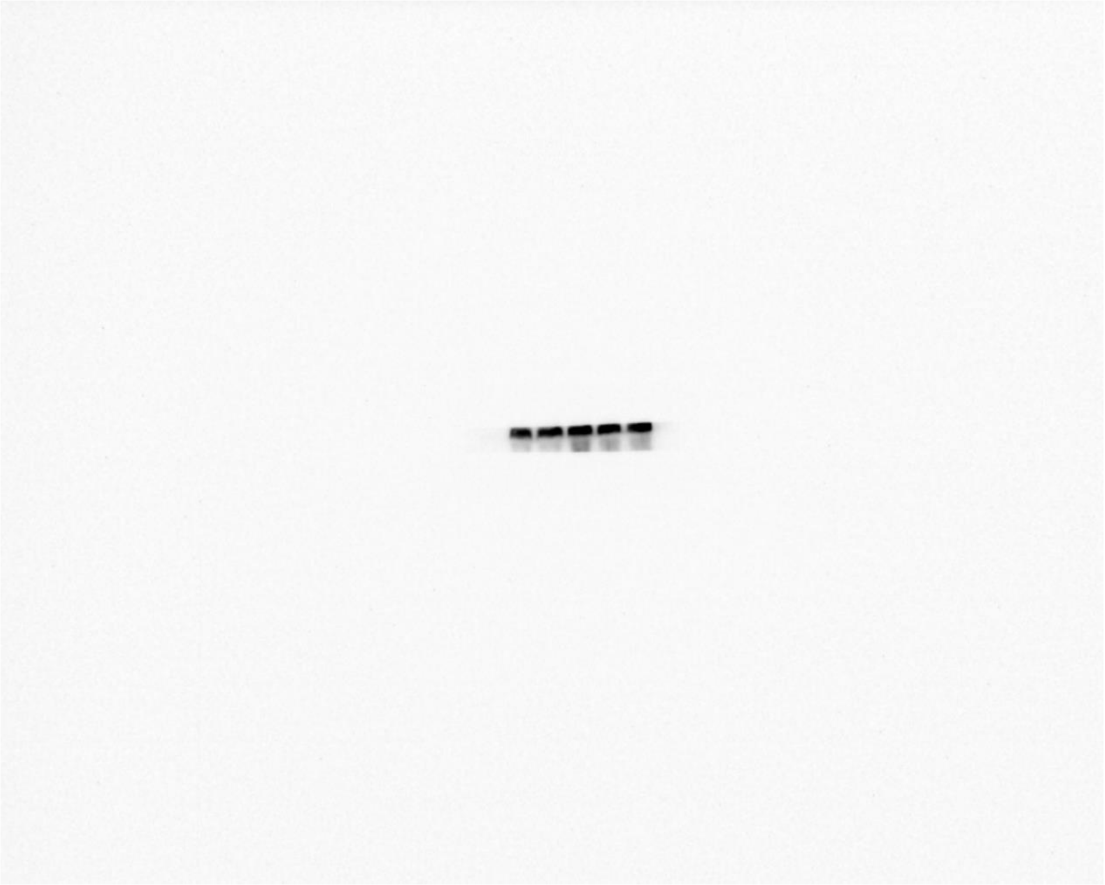

Figure S4 Un-cropped IκBα.

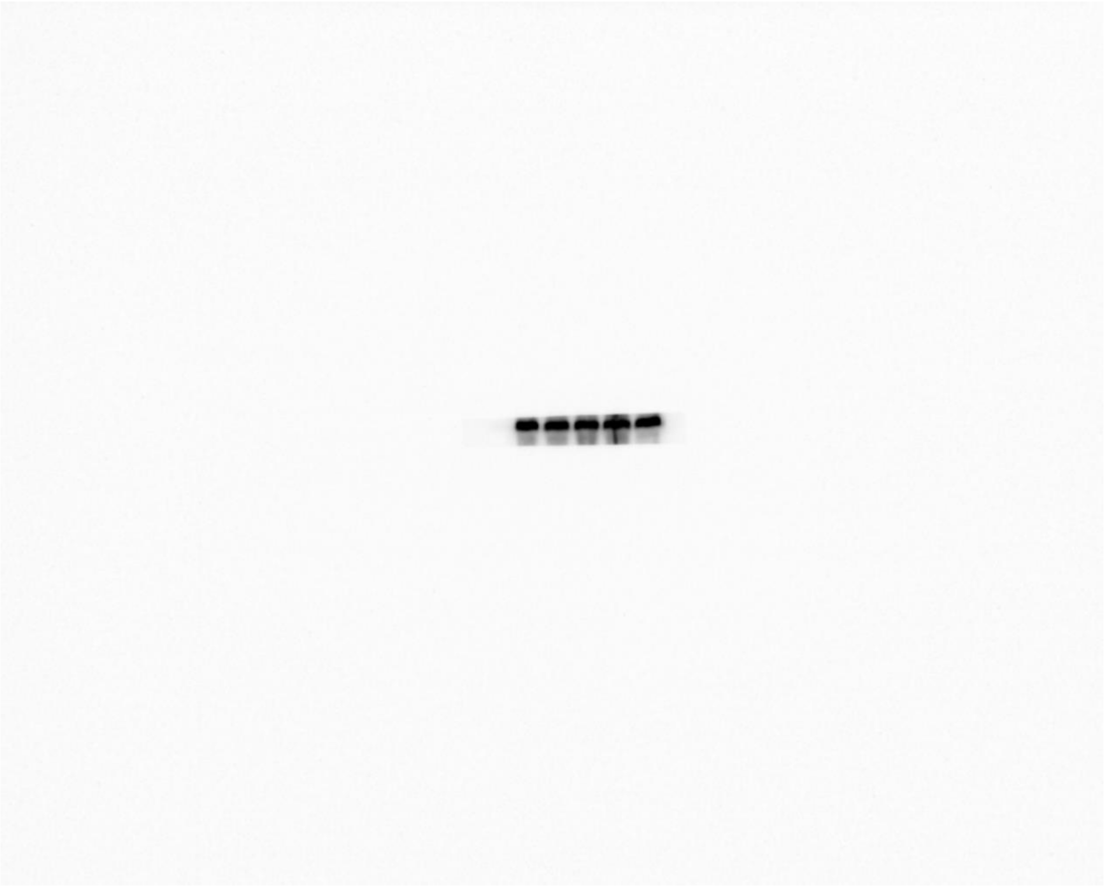

Figure S5 Un-cropped *β*-actin.

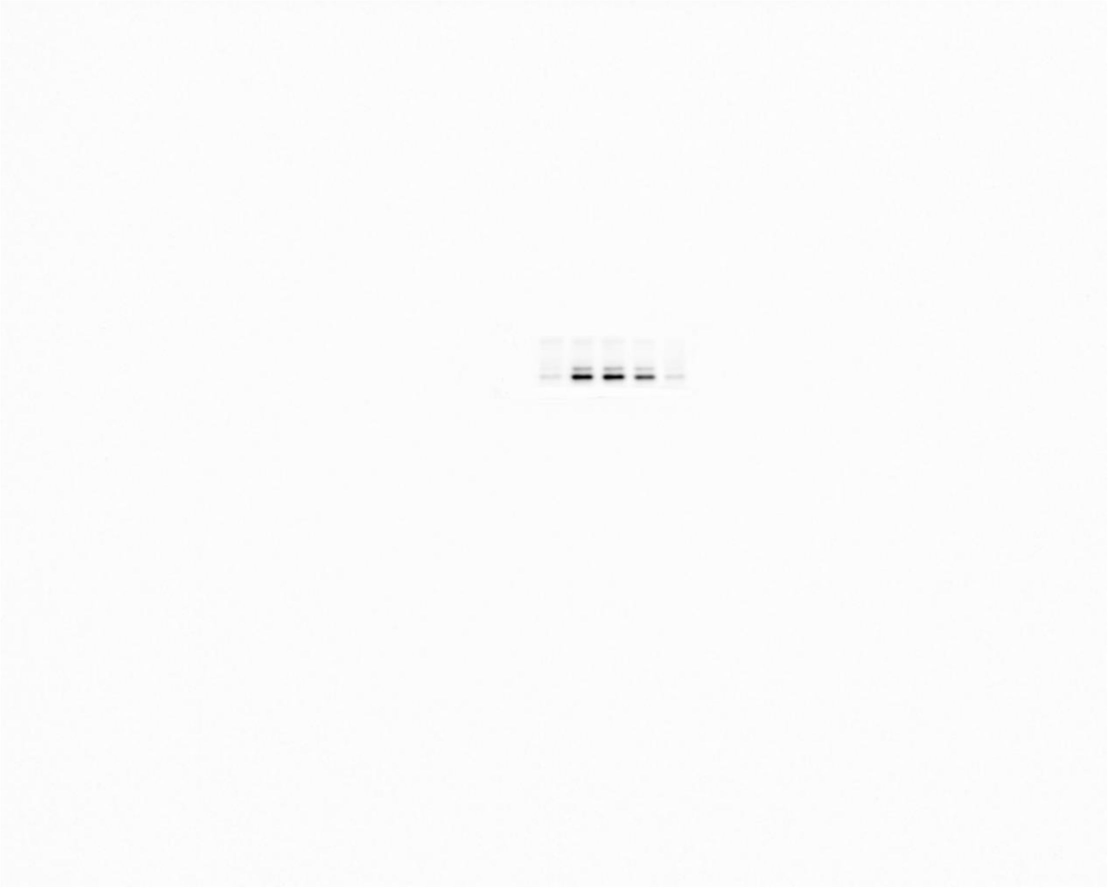

Figure S6 Un-cropped *p*-ERK1/2.

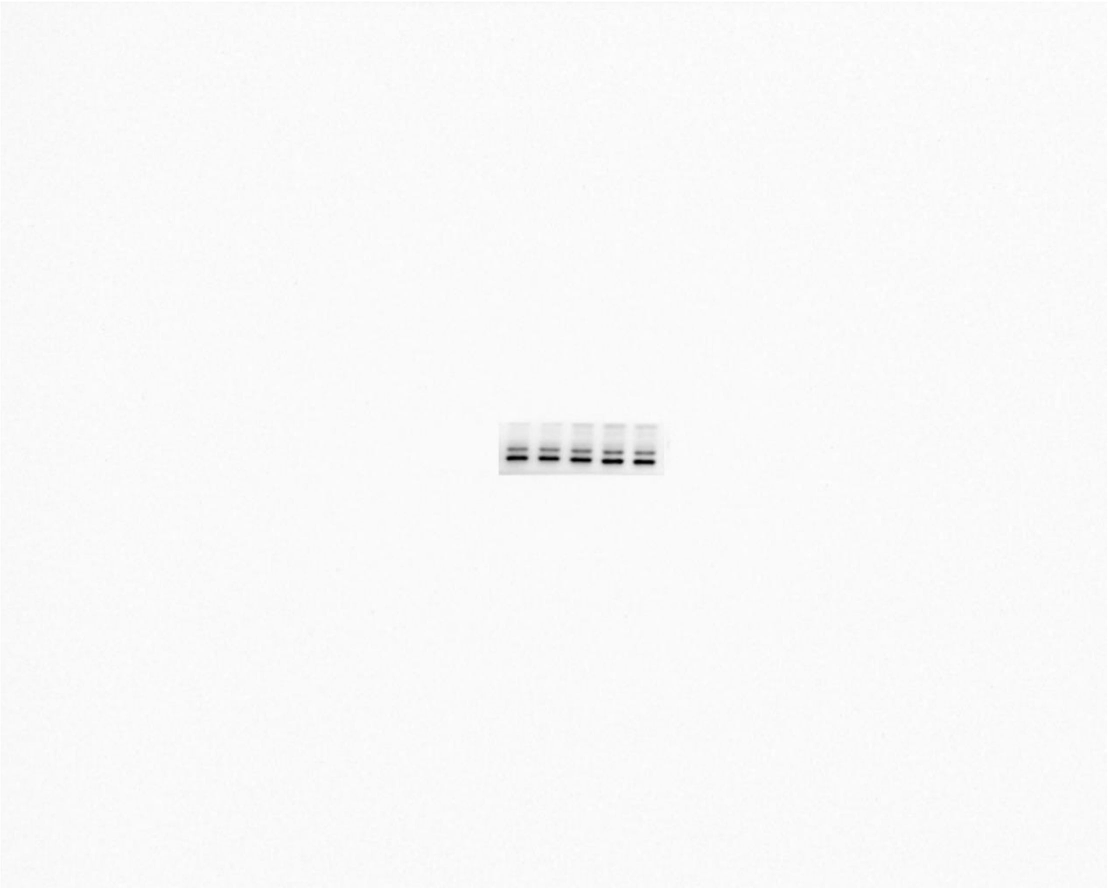

Figure S7 Un-cropped ERK1/2.

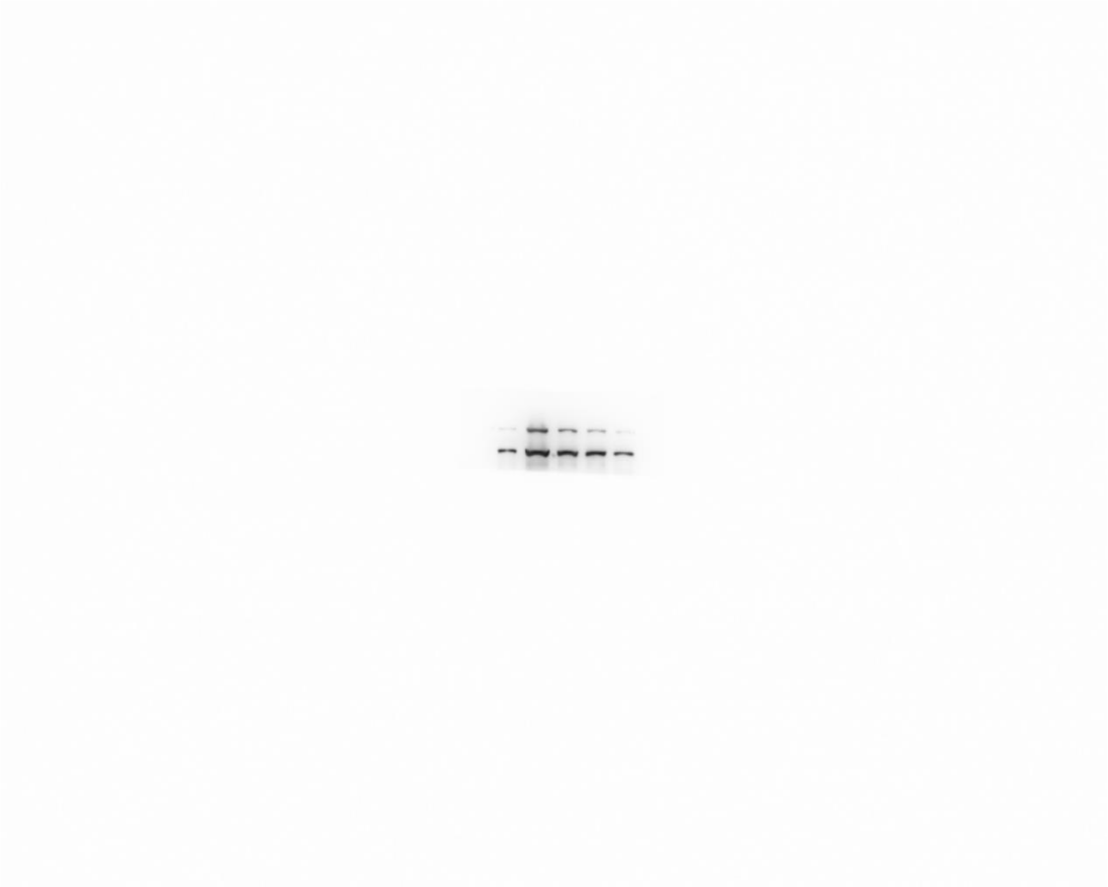

Figure S8 Un-cropped *p*-JNK.

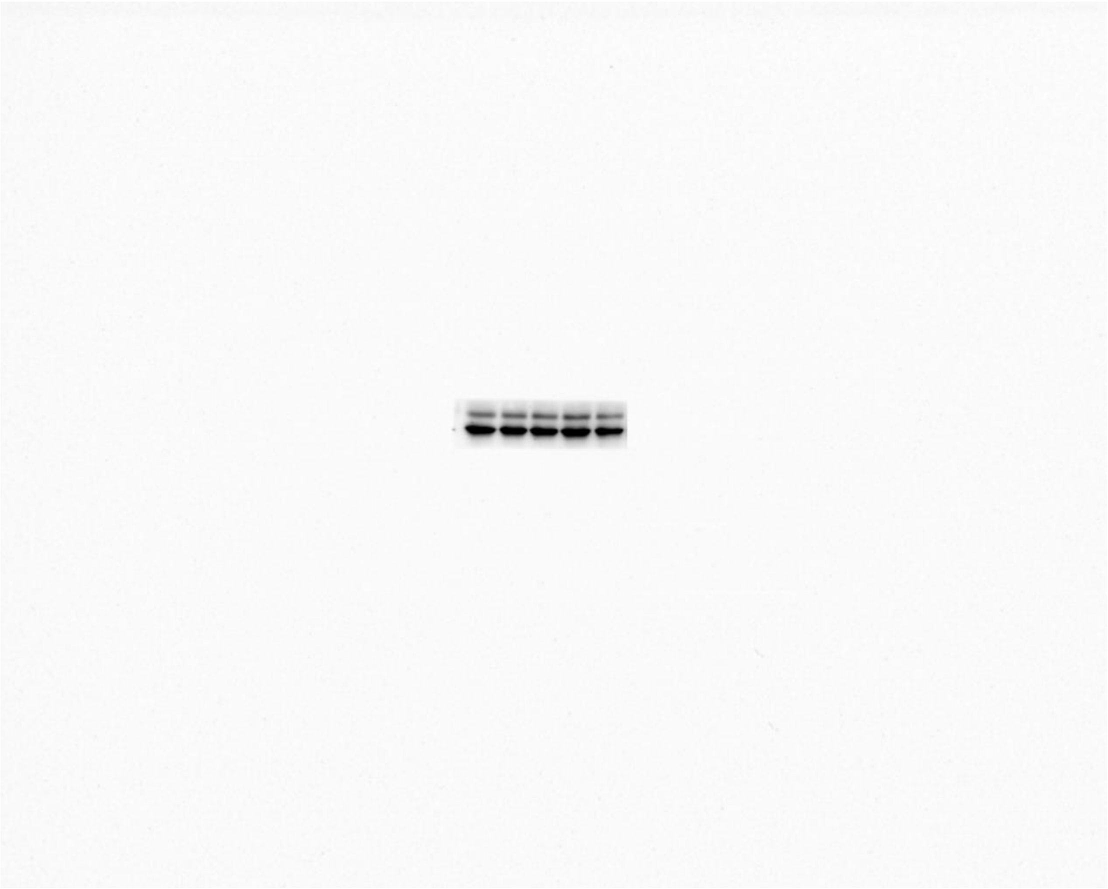

Figure S9 Un-cropped JNK.

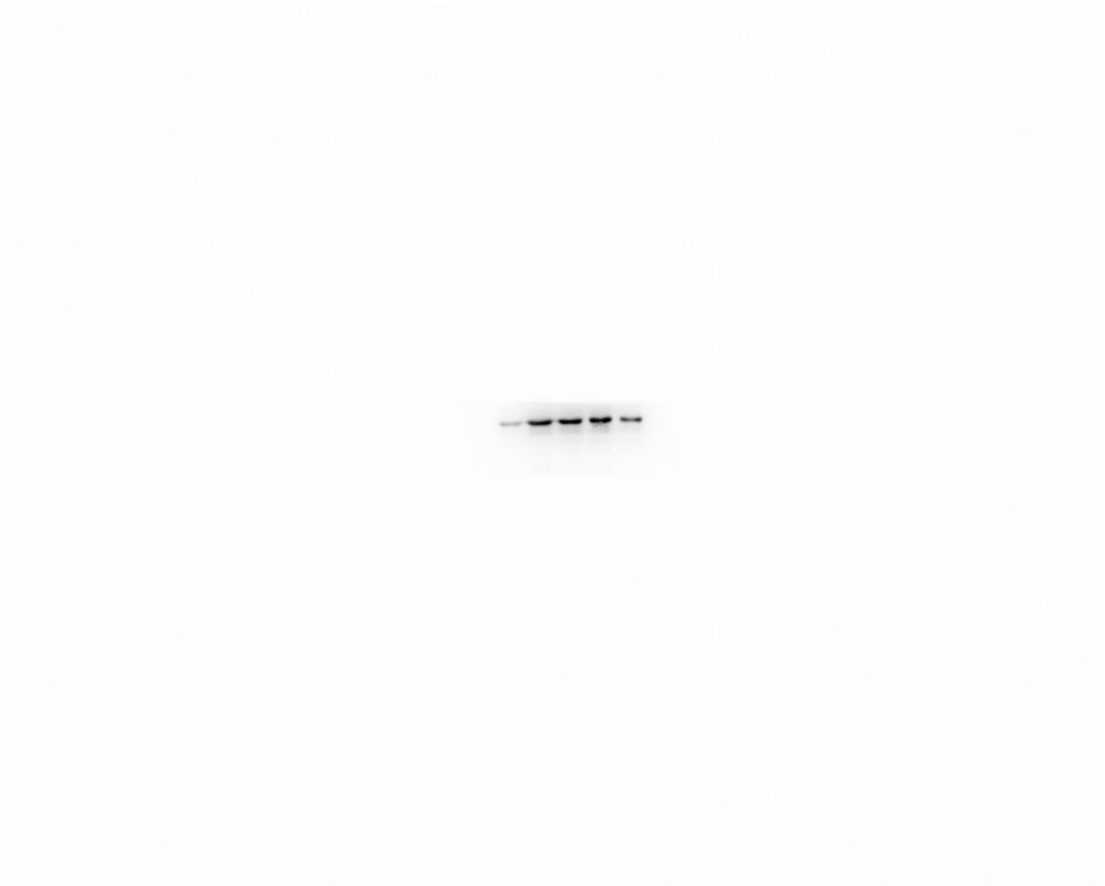

Figure S10 Un-cropped *p*-p38.

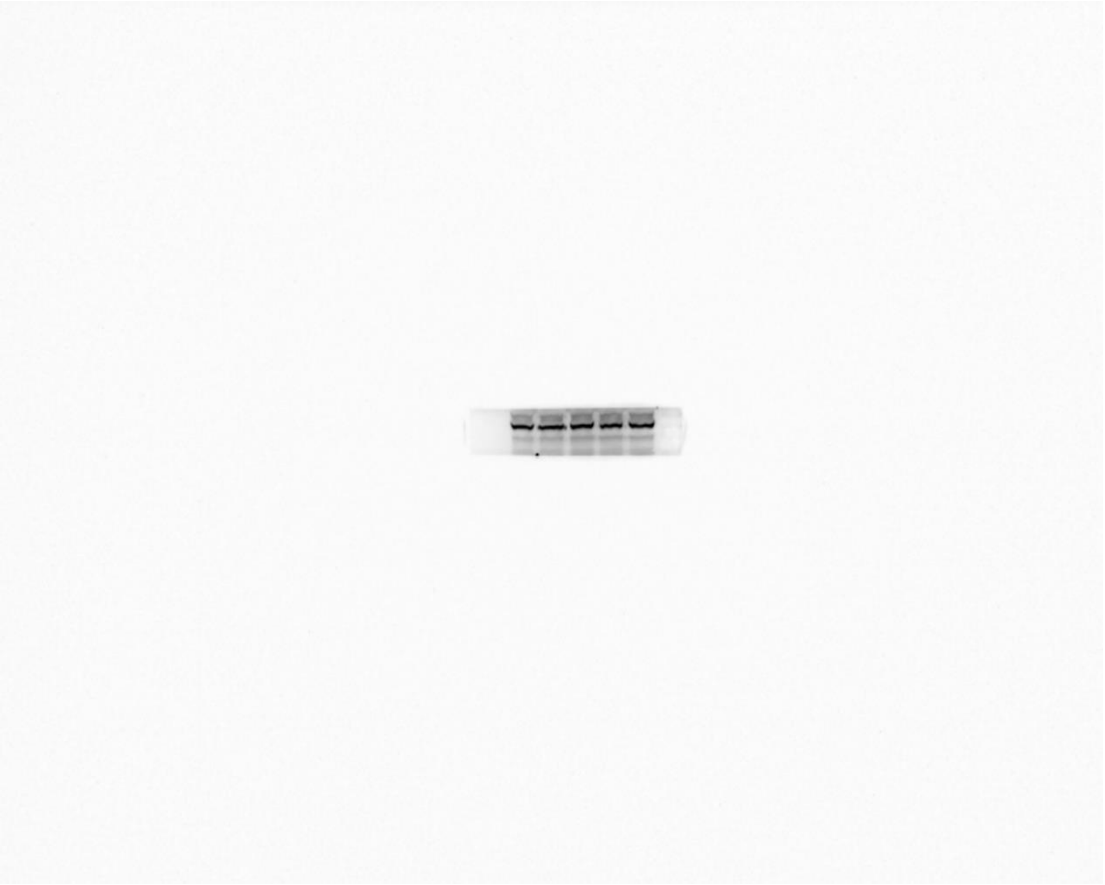

Figure S11 Un-cropped p38.

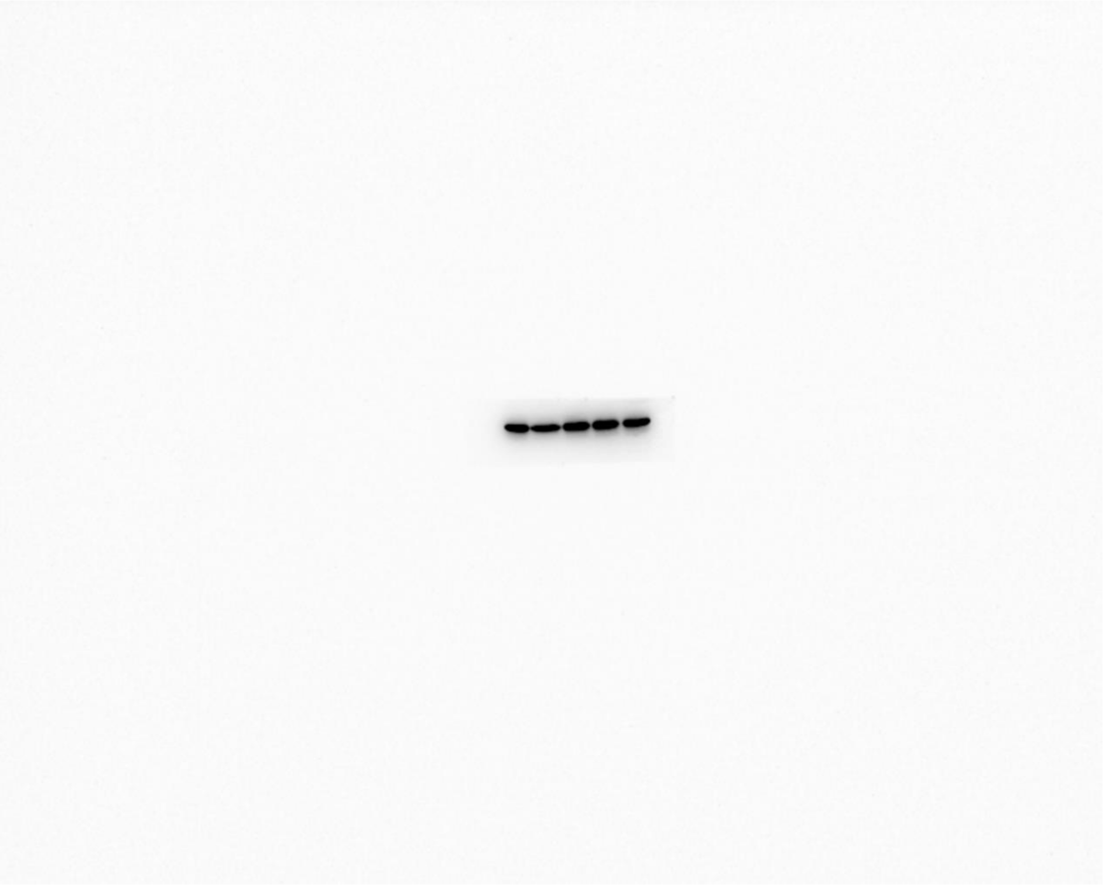

Figure S12 Un-cropped *β*-actin.
